# Novel Y Chromosome Retrocopies in Canids Revealed through a Genome-Wide Association Study for Sex

**DOI:** 10.3390/genes10040320

**Published:** 2019-04-25

**Authors:** Kate L. Tsai, Jacquelyn M. Evans, Rooksana E. Noorai, Alison N. Starr-Moss, Leigh Anne Clark

**Affiliations:** 1 Department of Genetics and Biochemistry, Clemson University, Clemson, SC 29634, USA; ktsai@clemson.edu (K.L.T.); jacquelyn.evans@nih.gov (J.M.E.); astarr@clemson.edu (A.N.S.-M.); 2 Cancer Genetics and Comparative Genomics Branch, National Human Genome Research Institute, National Institutes of Health, Bethesda, MD 20892-2152, USA; 3 Clemson University Genomics and Bioinformatics Facility, Clemson University, Clemson, SC 29634, USA; rooksan@clemson.edu

**Keywords:** dog, canine, *MITF*, *PPP2CB*, *WNK1*, *ETV5*, retrogene

## Abstract

The lack of an annotated reference sequence for the canine Y chromosome has limited evolutionary studies, as well as our understanding of the role of Y-linked sequences in phenotypes with a sex bias. In genome-wide association studies (GWASs), we observed spurious associations with autosomal SNPs when sex was unbalanced in case-control cohorts and hypothesized that a subset of SNPs mapped to autosomes are in fact sex-linked. Using the Illumina 230K CanineHD array in a GWAS for sex, we identified SNPs that amplify in both sexes but possess significant allele frequency differences between males and females. We found 48 SNPs mapping to 14 regions of eight autosomes and the X chromosome that are Y-linked, appearing heterozygous in males and monomorphic in females. Within these 14 regions are eight genes: three autosomal and five X-linked. We investigated the autosomal genes (*MITF*, *PPP2CB*, and *WNK1*) and determined that the SNPs are diverged nucleotides in retrocopies that have transposed to the Y chromosome. *MITFY* and *WNK1Y* are expressed and appeared recently in the Canidae lineage, whereas *PPP2CBY* represents a much older insertion with no evidence of expression in the dog. This work reveals novel canid Y chromosome sequences and provides evidence for gene transposition to the Y from autosomes and the X.

## 1. Introduction

The Y chromosome is historically described as a degenerate X chromosome that has lost about 95% of its ancestral genes and is poorly conserved among mammals [1,2]. Evolutionary compensation for gene loss on the Y is proposed to occur through the transposition and retrotransposition of genes from the Y chromosome to other chromosomes [3,4]. In the opposite direction, the Y chromosome has acquired new genetic material from the X chromosome or autosomes through the same mechanisms [5,6,7,8,9]. Novel Y-linked genes usually have testis-specific expression and function in spermatogenesis, likely conferring a reproductive advantage in males [1,8,10,11].

Sequence for the Y chromosome is not readily available for many organisms that have an otherwise complete reference genome. The 7.6X canine reference genome was generated from a female boxer, with >98% of assembled sequence ordered and oriented on 39 nuclear chromosomes and 3228 unplaced scaffolds [12,13]. Having been excluded from the reference genome, ascertainment of canine Y chromosome sequences has been the focus of subsequent studies. Approximately 24 kb of Y chromosome sequences were determined through the identification of shotgun reads that 1) possessed sequence similarity to the human Y chromosome and 2) were present in a 1.5X sequence from a male poodle, but not the female reference genome [14]. A 2013 study of the canine Y chromosome utilized expressed sequences and a Doberman BAC library to capture novel Y sequences and annotate male-specific genes, resulting in the most complete Y chromosome reference sequence available to date [15].

Today, commercially-available canine SNP arrays include Y chromosome markers; however, the arrays were initially developed using highly polymorphic SNPs identified from the reference genome and lower coverage sequences (~1–1.5X) from 10 dog breeds, 4 wolves, and a coyote [12]. Genomes from male dogs sequenced for SNP discovery included reads from the Y chromosome, which presumably remained unmapped. While performing genome-wide association studies (GWASs) for complex traits using SNP data generated from the Illumina 230K CanineHD array, we observed spurious associations with autosomal SNPs when sex was not balanced between cases and controls. This led us to hypothesize that some SNPs placed on autosomes are sex-linked. Here, we test our hypothesis by performing a GWAS for sex.

## 2. Materials and Methods

Genomic DNAs and tissues used for this work were obtained with protocols approved by the Clemson University Institutional Review Board (IBC2008–17). Genotyping was performed for 72 German shepherd dogs using the Illumina CanineHD 230K BeadChip. SNPs having call rates <95% and minor allele frequencies <5% were removed, leaving 123,247 SNPs. We used 25 females and 25 males having call rates >97% in a case-control analysis. Fisher’s exact *P*-values were calculated under a dominant model. Filtering and statistical analyses were performed with SNP & Variation Suite v8 (SVS, Golden Helix, Bozeman, MT).

Whole genome sequences from domestic dogs were available in our lab; all other Canidae genomes (Table S1) were downloaded from NCBI’s SRA database using fasterq-dump from sratoolkit v2.9.2 (https://ncbi.github.io/sra-tools/). The quality of reads was assessed by FastQC v0.11.6 (https://www.bioinformatics.babraham.ac.uk/projects/fastqc/) before and after trimmomatic v0.36 [16] was run to remove low quality bases and adapter sequences. Gsnap v2018-05-30 [17] was used to align fastq files to the canFam 3.1 genome using default parameters. Samtools v1.6 [18] was used to convert, sort, and index the alignment files for review in Integrative Genomics Viewer (IGV) v2.4.14 [19]. Using IGV, we searched for evidence of the Y chromosome duplications and, in the autosomal expressed regions, we evaluated at least one male and one female from each species. When we observed characteristics of a duplication in the male, but not the female, and identified more than 2 shared polymorphisms with the *Canis familiaris* Y chromosome copy, we posited that an ancestral version of the retrocopy was present. Retrocopies and sequences from unmapped mate pairs were analyzed for similarities with the canine Y assembly (GenBank: KP081776.1) using NCBI BLAST.

To check for expression and splicing activity, we utilized archival heart, brain, and liver tissues obtained from intact male dogs. Total RNA was isolated using the ToTALLY RNA Total Cellular RNA Isolation kit (Ambion, Foster City, CA). We obtained dog testis RNA from Zyagen (San Diego, CA). To remove and digest genomic DNAs, the RNAs were treated using the TURBO DNA-free kit (Invitrogen, Carlsbad, CA), following the manufacturer’s instructions for rigorous treatment. cDNAs were synthesized with an oligo dT primer using the RevertAid First Strand cDNA Synthesis kit (Thermo Scientific, Pittsburgh, PA). For each tissue, a genomic control was run, to which no reverse transcriptase was added.

Primers to amplify the *MITF*, *PPP2CB*, and *WNK1/ETV5* Y chromosome retrocopies were designed in diverged sequences, when possible, and with forward and reverse primers in different exons. Primer sequences were: *MITFY*: F 5′-CAGGTGCCGATGGAAGT, R 5′-TGAAGGAGGTCTTGGTTGCT; *PPP2CBY*: F 5′-CTGAACGAGAACCAAGTGT, R 5′-CACCAAATAGGATGTAATGACAA; *WNK1Y*: F 5′-CTTTCAAGGAGAAGAAGGCG, R 5′-CAACCTGTCCCACTCTACTGAT. A fourth primer set uses an *ETV5Y* F (5′-CTTTCTGTCAATCACATGCCA) with *WNK1Y*-R. Primer sets to amplify a total of 1,879 bp of chrUn_AAEX03024224 were: ChUn1: F 5′-GTGGTGCTGACCCTGTGC; R 5′-TTCCCTGGTATGAGTGTCTGTG; ChUn2: F 5′-TGGGTGATTTGTAGATGGTAGG; R 5′-GGGTTGAGGGAATGGTGAA; ChUn3: F 5′-GAGGGTGGAGGGTCTGCT; R 5′-AACACAAGGGTAAATCAGGTCAC. Genomic DNAs from three female and three male German shepherd dogs were used to test for male-specific amplification. All PCRs were performed using Phire Green Hot Start II DNA Polymerase mix (Thermo Scientific) following manufacturer guidelines for a 20 μL reaction. Products were cleaned with ExoSAP-IT PCR Product Cleanup (Applied Biosystems, Foster City, CA), and sequence was confirmed using Sanger sequencing.

## 3. Results

**Figure 1:**
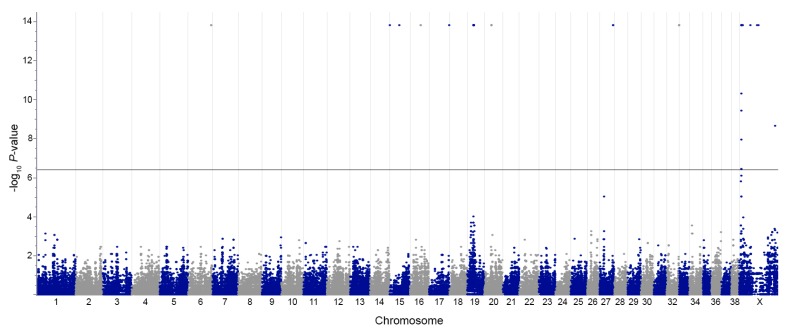
Manhattan plot illustrating results for a genome-wide association study for sex. The -log_10_ *P*-value (y-axis) is plotted against chromosome position (x-axis). The black horizontal line marks the threshold for Bonferroni significance.

A GWAS for sex identified 50 genome-wide significant SNPs with identical *P*-values of 1.58 × 10^−14^, mapping to eight autosomes and both sex chromosomes (Table 1). A Manhattan plot of the results shows no evidence of linkage disequilibrium decay at the identified loci, usually illustrated by proximal SNPs with increasingly larger *P*-values supporting the lead SNP, except in the pseudoautosomal region of the X chromosome (Figure 1). Inspection of genotypes for the 50 SNPs revealed all males to be heterozygous and all females to be monoallelic, except for chrX:60395963 in *PGK1* for which some males were homozygous. This SNP was excluded from further study. Of the remaining 47 non-Y chromosome SNPs, 19 are located within seven genes, 22 are intergenic, five are telomeric, and one is centromeric.

**Figure 2:**
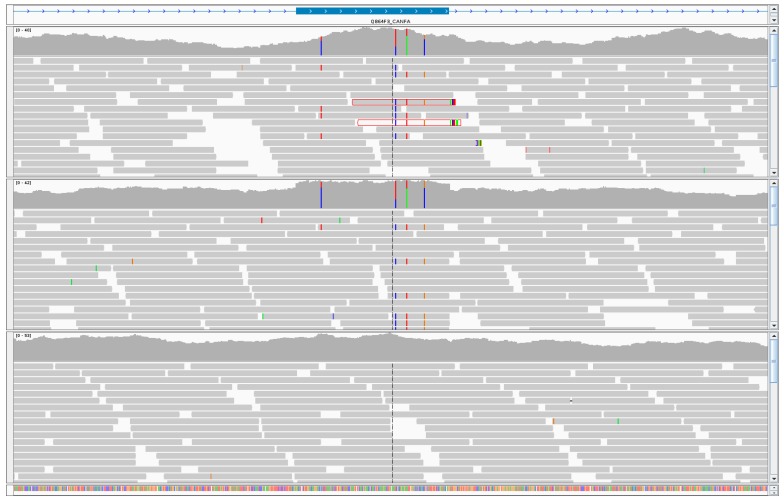
Integrative Genomics Viewer screenshot of a *MITF* exon illustrating increased read coverage, unmapped mate pairs (red outline), low mapping score (white read), and increased heterozygosity in male dogs (top and middle) as compared to a female (bottom).

**Figure 3:**
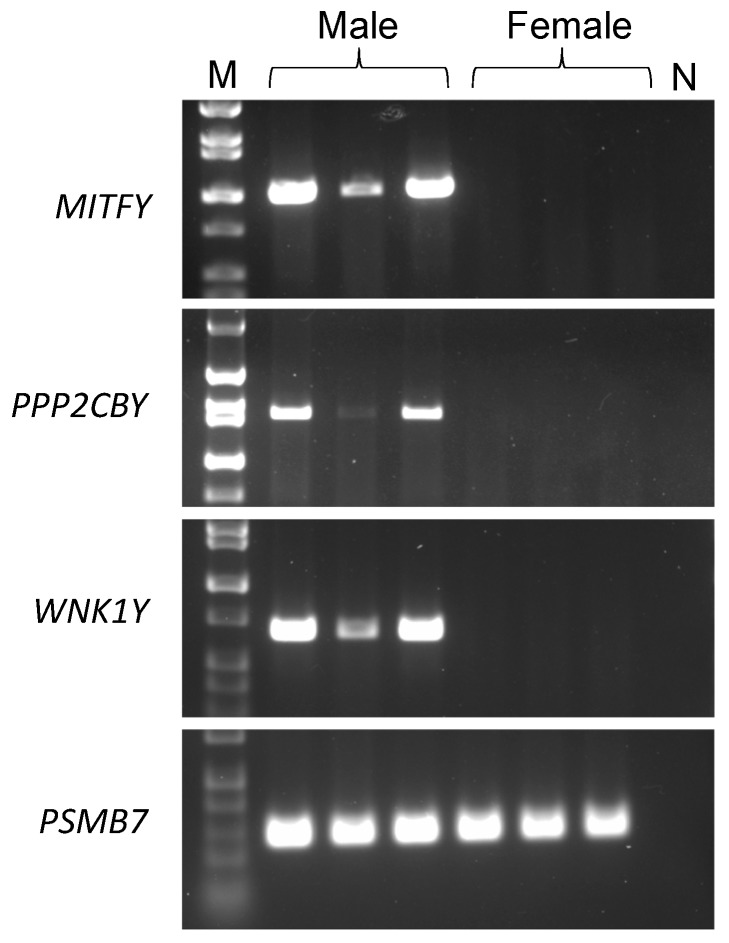
Primers were designed to detect Y-linked retrocopies of three genes, as well as the presence of a control autosomal gene (*PSMB7*). Polymerase chain reaction using DNA from 3 male dogs and 3 female dogs showed male specific amplification for the 3 retrocopies (top three panels). Amplification for all dogs is shown for *PSMB7* in the bottom panel.

We further investigated the SNPs mapping to exonic or untranslated regions of the autosomal genes *MITF*, *PPP2CB*, and *WNK1*. Using next generation sequencing reads aligned to the reference genome, we visually inspected each gene. Within the exons of male dogs only, we observed: distinct increases in sequence coverage, heterozygosity, unmapped mate pairs, and reads with low mapping scores, an indication that they map to multiple locations (Figure 2). Taken together, these findings led us to posit that the SNPs are present on diverged retrocopies located on the Y chromosome. To test our hypothesis, we used next generation sequencing data to design PCR primers that spanned several exons, such that only a retrocopy would amplify from genomic DNA. PCR with gDNA showed male-specific amplification for each of the three genes (Figure 3), confirming their location on the Y chromosome. We termed the three retrocopies: *MITFY*, *PPP2CBY*, and *WNK1Y*. The retrocopies and flanking sequences (determined using unmapped mate pairs) showed no significant similarities to the canine Y assembly.

### 3.1. MITFY

**Figure 4:**
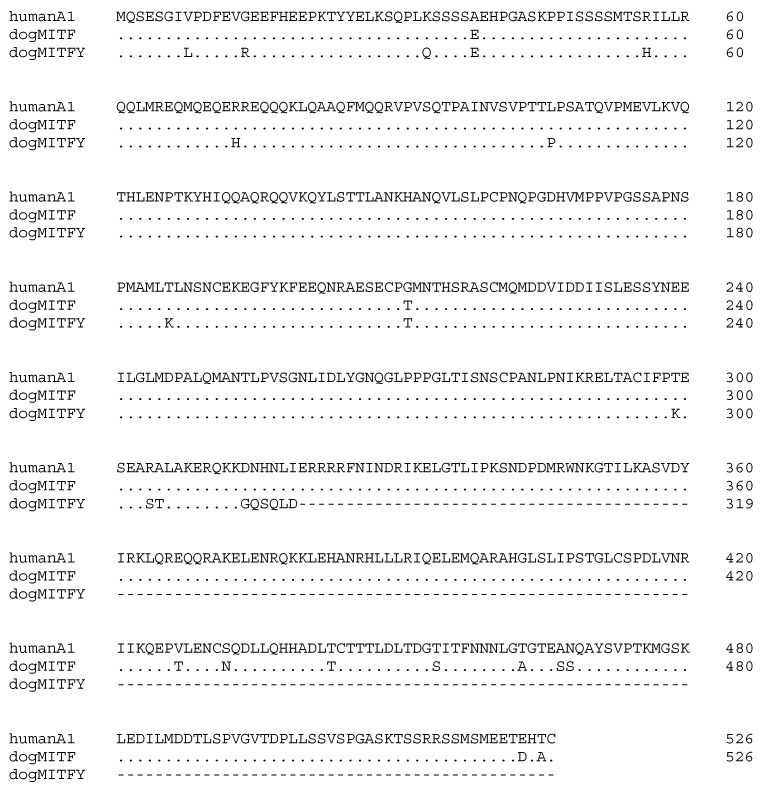
Alignment of MITF protein sequences: Human isoform A1, canine autosomal (CFA20), and predicted Y retrogene sequences.

We Sanger sequenced 944 bp of *MITFY* from genomic DNA and used overlapping next-generation sequencing reads to determine the 5′ and 3′ sequences. Parental *MITF*, located on CFA20, and *MITFY* are 98% identical (Figure S1). The retrocopy is consistent with human *MITF* isoform A1, which uses exon 1A and encodes a 526 aa protein. However, *MITFY* harbors a single base insertion that predicts a frameshift and premature stop codon that would truncate the protein by nearly 40% (Figure 4).

The *MITFY* primer set amplified transcripts arising from both the parental gene and retrogene. cDNA amplicons were obtained from all four tested tissues, sequenced, and genotyped for Y-specific variants. In testis, we detected expression of *MITF* and *MITFY*, evidenced by heterozygosity for Y chromosome and autosomal alleles. Heart, liver, and brain only showed expression of the autosomal copy.

**Figure 5:**
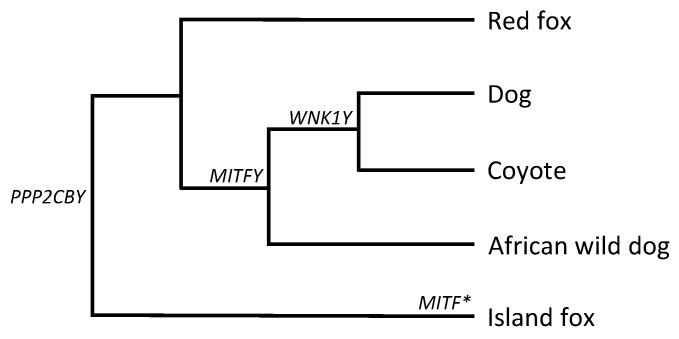
Cladogram (based on [12]) depicting the most parsimonious hypothesis for the retrotransposition of three genes in the Canidae family. *The *MITF* retrocopy in the island fox could not definitively be placed on the Y chromosome.

*MITF* retrogene sequences were also identified in WGS from males of older Canidae family members: Coyote (*Canis latrans*), African wild dog (*Lycaon pictus*), and island fox (*Urocyon littoralis*) (Figure 5). Red foxes (*Vulpes vulpes*) did not show evidence of a retrogene based on WGS. The domestic dog, coyote, and African wild dog shared a majority of *MITFY* variants, while the island fox possessed a unique variant haplotype.

### 3.2. PPP2CBY

We Sanger sequenced ~1330 bp of *PPP2CBY* from genomic DNA and determined the 5′ and 3′ sequences using WGS data. *PPP2CBY* is 89% identical to parental *PPP2CB*, located on CFA16 (Figure S2). The reverse *PPP2CBY* primer contains a 9 bp Y-specific deletion, and thus PCR was specific to the Y chromosome copy in both gDNA and cDNA. We found no evidence of expression of *PPP2CBY* in any of the four tissues examined and posit that it is a pseudoretrogene. Genomic evidence of *PPP2CBY* was present in all male (but not female) canid genomes investigated herein (Figure 5).

### 3.3. WNK1Y

We Sanger sequenced 668 bp of *WNK1Y* from genomic DNA and used overlapping next-generation sequencing reads to determine the 5′ and 3′ sequences of the retrocopy. The *WNK1Y* retrocopy has no identifiable translational start site and is composed of exons 25, 26, and a spliced version of exon 27. *WNK1Y* primers amplified transcripts arising from both the parental gene and retrogene. cDNA amplicons were obtained from testis and heart tissues. Sequencing of amplicons showed that *WNK1Y* transcripts were present in both tissues.

Using BLAT, we discovered that all 1041 bp of *WNK1Y* align with 95.4% similarity to a continuous fragment of an unplaced scaffold: chrUn_AAEX03024224 (chrUn hereafter). Our original *WNK1Y* primer set, used to show male-specific amplification, did not amplify the chrUn sequences because of diverged nucleotides in the primer sequences. We designed new primers sets within and flanking the *WNK1* sequences on chrUn that amplified in both males and females (data not shown), thereby confirming that the chrUn retrocopy is autosomal. Sequence alignment of the chr27 parent *WNK1*, *WNK1Y*, and chrUn *WNK1* revealed five shared SNPs between the two retrocopies (Figure S3). Using IGV, we observed signatures of a Y chromosome haplotype beyond *WNK1*, across approximately 6500 bp of the 9600 bp chrUn scaffold, in both dog and coyote.

We also observed many chrUn *WNK1* reads having mate pairs mapping to CFA34, corresponding to *ETV5*. We visually inspected WGS flanking *WNK1* and identified an *ETV5* retrocopy containing exons 3, 4, and 5 and partial intron 5. *WNK1* and *ETV5* are arranged in a head-to-head orientation. Using an *ETV5* forward primer designed using changes on the Y haplotype and a *WNK1Y* reverse, we observed male-specific amplification (data not shown), indicating that like *WNK1*, *ETV5* retrocopies exist on both chrUn and the Y chromosome. The primers did not yield amplicons from cDNA.

## 4. Discussion

A GWAS revealed 50 SNPs significantly associated with sex, 49 of which are in perfect linkage disequilibrium, despite mapping to 10 different chromosomes. Based on the data presented here, we posit that these SNPs represent autosome or X chromosome sequences that are duplicated on the Y chromosome. Both sexes possess two wild-type copies, while males also have a diverged Y-chromosome copy for which they are hemizygous. When aligned to the female reference genome, the Y-chromosome sequences align with the homologous sequences, making the diverged nucleotides appear to be SNPs that are highly polymorphic across breeds and other canids. Thus, 47 of the 49 Y-linked SNPs revealed in our GWAS were misplaced on the genome map. The two SNPs correctly placed on the Y chromosome likely have a homologous autosomal or X chromosome sequence, causing monomorphic genotypes in SNP array data from females.

In this study, we focused on the Y-linked SNPs that map to an autosomal gene and discovered novel canid retrocopies, two of which show transcriptional activity. In dogs, two other expressed retrogenes have been associated with common skeletal malformations; both originate from fibroblast growth factor 4 (*FGF4*) on CFA18 [20,21,22]. Additional studies based on the Ensembl annotation of the canine reference genome suggest that there are between 95 and 409 retrogenes in dogs, but none are confirmed to be expressed other than the *FGF4* retrogenes [23,24,25].

Here, we confirmed transcription of *MITFY* in testis, although it is not clear whether the retrogene produces a functional protein. We found no evidence for novel introns within *MITFY*, thus the premature stop codon is unlikely to trigger nonsense mediated decay if it is translated [26,27]. The parental gene, *MITF*, encodes a transcription factor involved in the development of various cell types, most notably melanocytes [28]. The predicted premature stop codon in the Y retrogene occurs within the bHLH-LZ domain, which would normally permit DNA binding of target genes [28]. However, exons 1A and 4, which enable transactivational activity [29], precede the *MITFY* translational termination signal. At the mRNA level, *MITF* is a known target of several miRNAs [30]. Though we observed numerous retrogene-specific variants in the 3′ UTR, we found that many miRNA bindings sites are preserved between parental *MITF* and the retrogene. Without additional studies, the activity of *MITFY* and its impact on the expression and/or function of parental *MITF* is unclear.

*WNK1Y* transcripts were present in testis and cardiac tissue. WNK1 is a serine-threonine kinase encoded by a 33 exon gene with multiple tissue-specific isoforms [31]. *WNK1Y* is composed of exons 25, 26, and parts of exon 27, a splicing pattern inconsistent with previously described isoforms and for which we cannot find a clear translational start site. Immediately upstream of *WNKY* is an *ETV5Y* retrocopy in reverse orientation. The parent gene, *ETV5*, is an ETS transcription factor important in morphogenesis and fertility [32]. ETS transcription factors have a role in oncogenesis and *ETV5* gene fusions have been described in prostate cancers [33]. *ETV5Y* consists of three exons, part of an intron, and lacks the translational start site. We also found *WNK1* and *ETV5* retrocopies in the same head-to-head orientation on an unplaced scaffold, chrUn_AAEX03024224. Examination of the remainder of the scaffold revealed a male-specific haplotype in the dog and coyote, suggesting that the unplaced scaffold is duplicated, at least in part, on their Y chromosomes.

Our data are inconclusive with regard to the chromosomal location of the island fox *MITF* retrocopy, which did not share variants with *MITFY*. Unfortunately, WGS from a female of the same subspecies was not available to eliminate or confirm the presence of an autosomal retrocopy event. Regardless, we hypothesize that the island fox retrocopy is an independent event and that *MITFY* arose after the divergence of the red fox. Independent retrocopy events stemming from the same parental gene have been reported numerous times [4,25,34]. Each of the parental genes of the retrocopies identified herein has at least one other retrocopy insertion event in another species [35,36]. *PPP2CB* retrogenes have been identified in several mammals, with some species having multiple copies and showing transcriptional activity [36]. That distinct lineages have had retrocopies arising from the same genes and independently driven to fixation suggests that these events may have conferred increased fitness.

Like *FGF4*, the parent genes of the novel retrocopies identified herein all have roles in spermatogenesis [29,37,38,39]. Studies in other organisms have similarly observed that parental genes often have male-specific functions [34,40]. It has been suggested that transcripts present during spermatogenesis may be more likely to become retrocopies because of the open configuration of chromatin during periods of high transcriptional activation [41]. This may also underlie the observation that retrogenes are often expressed in testes [41], as we observed with *MITFY* and *WNK1Y*.

We did not find evidence for novel X chromosome derived retrogenes. Two of the Y-linked SNPs mapped to the X chromosome are in a previously described 120 kb X to Y chromosome transposition event in dogs that includes *TRAPPC2* and *OFD1* [15]. Manual inspection in IGV confirmed the presence of this duplication in all canid sequences investigated herein. A duplication of *OFD1* and portions of *TRAPPC2* and *GPM6B* has been described in pigs, with high *OFD1Y* expression observed in porcine testis [42], and *OFD1* transposition to Y has also been described in primates [43]. The frequent transposition of *OFD1* to the Y chromosome in mammals may indicate an acquired function in spermatogenesis [42]. A sex-linked Illumina SNP was also observed in *SHROOM2*, located in the pseudoautosomal region of the X chromosome [15]. We detected both exonic and intronic variants on a shared male-specific haplotype, suggesting that like *OFD1*, the entire gene has been duplicated onto the canid Y.

## 5. Conclusions

We have shown that 47 SNPs from the Illumina CanineHD 230K are actually variations in homologous sequences located on the Y chromosome. This work illustrates that retrocopies and other genetic duplications are a source of false variant calls, a complication of using short read technologies and/or having an incomplete reference genome. We further investigated 12 SNPs mapping to expressed autosomal sequences and identified four novel canid Y chromosome retrocopies, two having transcriptional activity. The remaining SNPs mark chromosomal segments that have likely been duplicated on the Y chromosome, including nearly 300 kb of chromosome 19 that harbors two lincRNAs. Additional research into these regions holds promise for the identification of unique Y chromosome sequences. Complete sequence and annotation of the canine Y chromosome would be a valuable resource for researchers of multifactorial traits, particularly those that show a sex predilection such as heart, autoimmune, and infectious diseases.

## Figures and Tables

**Table 1:** Genome-wide significant markers identified in a GWAS for sex (*P*-value = 1.58 × 10^−14^).

Chr	Position	Gene	Region	Chr	Position	Gene	Region
6	72952463			20	21870155	*MITF*	exonic
	72964379				21870230	*MITF*	exonic
15	111194				21870310	*MITF*	exonic
	29973349				21870623	*MITF*	3′ UTR
16	33566554	*PPP2CB*	3′ UTR		21871904	*MITF*	3′ UTR
	33566935	*PPP2CB*	3′ UTR		21872335	*MITF*	3′ UTR
17	64209287				21872815	*MITF*	3′ UTR
19	20034966				21873532	*MITF*	3′ UTR
	20040153			27	42911747	*WNK1*	exonic
	20052361				42911917	*WNK1*	exonic
	20062503			32	38734101		
	20072295				38767343		
	20100030				38788489		
	20114792				38789367		
	20130159			X	6604781	*SHROOM2*	exonic
	20152245				6621021	*SHROOM2*	intronic
	20172164				6628533	*SHROOM2*	intronic
	20245553				6634742	*SHROOM2*	intronic
	20256174				10131021	*TRAPPC2*	exonic
	20286580				10175834	*OFD1*	exonic
	20292945				35604689	*USP9X/Y*	exonic
	20303661				57139861		
	20309777				60395963	*PGK1*	
	20310190			Y	26641		
	20314276				316950		

## Supplementary Materials

The following are available online at https://www.mdpi.com/2073-4425/10/4/320/s1, Figure S1: title, Table S1: title, Video S1: title. The following are available online: Figure S1: Alignment of *MITFY* and parental *MITF* (Chr20) sequences, the start and stop codons are bolded, Illumina SNPs are highlighted in yellow; Figure S2: Alignment of *PPP2BCY* and parental *PPP2CB* (Chr16) sequences, the start and stop codons are bolded, Illumina SNPs are highlighted in yellow; Figure S3: Alignment of *WNK1* sequences: parent gene (Chr27), Y chromosome retrogene, and chrUn_AAEX03024224 retrocopy, Illumina SNPs are highlighted in yellow; Table S1: Sequence Read Archive accession numbers for resequencing data used herein.
