# Supplementary material for: Novel Y Chromosome Retrocopies in Canids Revealed through a Genome-Wide Association Study for Sex
Source: Genes (Basel). 2019 Apr 25;10(4):320. doi: 10.3390/genes10040320 (PMC6523286; doi:10.3390/genes10040320)
Supplement: Supplementary file 1 [file genes-10-00320-s001.zip › Supplementary figures/Fig S1.pdf]

Figure S1

MITF	CCCCCCGCCCCGGGCTCTGTTCTCACTTTGCAGCCGTGGAAGGACTGGGAGCGGGAGCC**A**	60
MITFY	A.....A....A..C.......	60
MITF	**TG**CAGTCGGAATCGGGGATCGTGCCGGATTTCGAAGTCGGGGAGGAGTTTCACGAAGAGC	120
MITFY	....................C.................A.....................	120
MITF	CCAAAACCTATTACGAACTCAAAAGTCAACCTCTGAAGAGCAGCAGTTCCGAGGAGCATC	180
MITFY	.......G...........................C.............T..........	180
MITF	CTGGGGCCTCCAAGCCTCCGATAAGCTCCTCCAGTATGACATCACGCATCTTGCTACGCC	240
MITFY	....A..............	240
MITF	AGCAACTCATGCGTGAGCAAATGCAGGAGCAGGAGCGCAGGGAGCAGCAGCAGAAGCTGC	300
MITFY	.A..................................A.......................	300
MITF	AGGCGGCCCAGTTCATGCAACAGAGAGTGCCTGTGAGCCAGACCCCAGCCATAAACGTCA	360
MITFY	....	360
MITF	GCGTGCCCACCACCCTTCCCTCTGCCACCCAGGTGCCGATGGAAGTCCTCAAGGTGCAGA	420
MITFY	.T.............C..	420
MITF	CCCACCTCGAAAACCCCACCAAGTACCACATACAGCAAGCCCAAAGGCAGCAGGTAAAGC	480
MITFY	....	480
MITF	AGTACCTTTCTACCACTTTAGCAAATAAACATGCCAACCAAGTCCTGAGCTTGCCATGTC	540
MITFY	....	540
MITF	CAAACCAGCCTGGCGATCATGTCATGCCACCAGTGCCGGGGAGCAGCGCACCCAACAGCC	600
MITFY	....T.............	600
MITF	CCATGGCTATGCTCACACTTAACTCCAACTGTGAAAAAGAGGGATTTTATAAGTTTGAAG	660
MITFY	...............A..	660
MITF	AGCAAAACAGGGCGGAAAGTGAATGCCCAACCATGAACACGCATTCACGAGCATCGTGCA	720
MITFY	....	720
MITF	TGCAGATGGATGATGTAATTGATGACATCATTAGCCTAGAATCAAGTTATAATGAAGAAA	780
MITFY	....	780
MITF	TCCTGGGATTGATGGATCCTGCTTTGCAAATGGCAAATACGTTACCTGTCTCTGGAAATC	840
MITFY	....	840
MITF	TGATTGATCTTTATGGCAACCAAGGCCTGCCTCCCCCAGGCCTCACCATCAGCAACTCCT	900
MITFY	....	900
MITF	GTCCAGCCAACCTTCCCAACATAAAAAGGGAGCTCACAGCGTGTATTTTTCCCACAGAAT	960
MITFY	....A.....	960
MITF	CTGAAGCGAGAGCATTGGCTAAAGAGAGGCAAAAAAA-GGACAATCACAACTTGATTGAA	1019
MITFY	..........TA.........................A......................	1020
MITF	CGAAGACGACGATTTAACATAAATGACCGCATTAAAGAACTAGGTACTTTGATTCCCAAG	1079
MITFY	.......................C....................................	1080

```
MITF    TCAAATGATCCAGACATGCGTTGGAACAAGGGAACCATCTTAAAAGCATCTGTGGACTAT 1139
MITFY   .A.................................T........................ 1140

MITF    ATCCGAAAGTTGCAACGAGAACAGCAACGTGCAAAAGAACTTGAAAATCGACAGAAGAAA 1199
MITFY   ...............................................C..........T. 1200

MITF    TTGGAGCACGCCAACCGGCATTTGTTGCTCAGAATACAGGAACTTGAAATGCAGGCTCGA 1259
MITFY   ...............G............................................ 1260

MITF    GCTCATGGACTTTCACTTATTCCATCCACGGGCCTCTGCTCTCCAGACTTGGTGAATCGG 1319
MITFY   ................................................A........... 1320

MITF    ATCATCAAGCAGGAACCCACTCTTGAGAACTGCAACCAAGACCTCCTTCAGCATCATGCA 1379
MITFY   ..............................A............................. 1380

MITF    GACCTACCTTGTACGACGACGCTTGATCTCACAGATGGCAGCATCACCTTCAACAACAAC 1439
MITFY   ...................................G........................ 1440

MITF    CTTGGAGCCGGGACCGAGAGTAGCCAAGCCTATAGCGTCCCCACGAAAATGGGATCCAAA 1499
MITFY   ...........................................T......T......... 1500

MITF    CTGGAAGACATCCTGATGGATGACACTCTTTCTCCCGTTGGTGTAACTGACCCACTCCTT 1559
MITFY   ............................................................ 1560

MITF    TCATCAGTGTCCCCTGGAGCTTCCAAAACAAGCAGCCGAAGGAGCAGCATGAGCATGGAA 1619
MITFY   ..........T................................................. 1620

MITF    GAAACCGATCATGCTTGTTAGCAGGCCCTCCCTGCTCTGCGCTTTCAAAAACTGCTTCCT 1679
MITFY   .....T...................................................... 1680

MITF    TTCTTGATTCGTAGGTTTCATAATTTACCTGAAGGGGTTTTCTTGATAATTTTCCTTTAA 1739
MITFY   .................................C.......................... 1740

MITF    TATGAAATTTTTTTTCATGCTTTACCAATAGCCCAGGATATATTTTATTTTTAGAATTTT 1799
MITFY   .....--...................................................G. 1798

MITF    GTGAAACAGACTTGTATATTCTATTTTACAACTACAAATGCCTCCAAAGTCTTGTACCAT 1859
MITFY   ........................G.............G..................... 1858

MITF    ACGTGTACAGTATCTGTGAACTGAATTCACCCTGGACTTTAGCTTTCTGAGCAAGAGGAT 1919
MITFY   .......................C.................................... 1918

MITF    TTTTGCATCAGAGAAATTTCTGTCCATTTTTATTCAGGGGAAACTTGGTTTGAGATTTTT 1979
MITFY   ..........................................G................. 1978

MITF    ATGTCTGTGGCATCTTTGGAAATTAAATGTAAAGTTTAATCGAAAGAATGTAAAGC     2035
MITFY   ........................................................     2034
```
