# Supplementary material for: Novel Y Chromosome Retrocopies in Canids Revealed through a Genome-Wide Association Study for Sex
Source: Genes (Basel). 2019 Apr 25;10(4):320. doi: 10.3390/genes10040320 (PMC6523286; doi:10.3390/genes10040320)
Supplement: Supplementary file 1 [file genes-10-00320-s001.zip › Supplementary figures/Fig S2.pdf]

Fig S2

PPP2CB	CCGCGCTCCCGCCCACCTCCCCGCGCCCTTCGCCGGCT----------------------	38
PPP2CBY	..A..........G......TG..-......A.T....CCCAGGGCGCGCGGCCGGGTGG	59
PPP2CB	GCG-GGGCGGGCGAGGGACCCCGCAGGCCGCGCGCCGGGAGCCGCCCGCTGGCCTTGGGC	97
PPP2CBY	...A..C..........T.T..........T...TG........................	119
PPP2CB	GGGGCGAGGGGCCCGCAGCCGCGCCGCCCCGCTCCCTCCTCTGCCGCTGGGAGCCGCTGG	157
PPP2CBY	C............T...A...C.T.............T...............T.....	179
PPP2CB	CGGCGGGAGAGGGGAGCGGCGCGCGGGGCGCGGGAGGGCAGGGGCGGGGTGGGGG-----	212
PPP2CBY	..................CG.G..C...T..............AT.....T....TGGTG	239
PPP2CB	--GAGGGAGGCAGCGGCCCGCC**ATG**GACGACAAGGCGTTCACCAAGGAGCTGGACCAGTG	270
PPP2CBY	GC.G........................C.....--------------------------	273
PPP2CB	GGTCGAGCAGCTCAACGAGTGTAAGCAGCTGAACGAGAACCAGGTGCGGACGCTGTGCGA	330
PPP2CBY	----...G....GG..C.........................A...T.C...........	329
PPP2CB	GAAGGCTAAGGAAATTTTAACAAAAGAATCAAATGTGCAAGAGGTTCGTTGTCCTGTAAC	390
PPP2CBY	...................T...................................A....	389
PPP2CB	CGTATGCGGAGATGTGCATGGTCAATTTCATGATCTTATGGAACTCTTTCGAATTGGTGG	450
PPP2CBY	.......A...........A........T...........A..T.....A..........	449
PPP2CB	AAAATCACCAGATACAAACTATCTGTTCATGGGCGACTATGTAGACAGAGGCTATTACTC	510
PPP2CBY	......-------.G...	502
PPP2CB	AGTGGAGACTGTGACTCTTCTTGTGGCATTAAAGGTGCGTTATCCAGAACGCATTACAAT	570
PPP2CBY	.........--........................G..............A.........	560
PPP2CB	ATTGAGAGGAAACCATGAAAGCCGACAAATTACCCAAGTATATGGCTTTTACGATGAATG	630
PPP2CBY	...............C.G...........C..T...........................	620
PPP2CB	T-CTACGAAAGTATGGAAATGCCAATGTTTGGAAATATTTCACAGATCTATTTGATTATC	689
PPP2CBY	.T...T..	680
PPP2CB	TTCCACTTACAGCTTTAGTAGATGGACAGATATTCTGCCTCCACGGTGGCCTCTCTCCAT	749
PPP2CBY	..........G.G..............GT...............................	740
PPP2CB	CCATAGATACACTGGATCATATCAGAGCTTTGGATCGTTTACAAGAAGTTCCACATGAGG	809
PPP2CBY	....T..A...............	800
PPP2CB	GCCCAATGTGTGATCTGTTGTGGTCAGATCCAGATGATCGTGGTGGGTGGGGTATTTCAC	869
PPP2CBY	....	860
PPP2CB	CTCGTGGTGCTGGCTACACATTTGGACAAGACATTTCTGAAACATTTAACCACGCCAATG	929
PPP2CBY	....C...G..	920
PPP2CB	GTCTCACACTGGTTTCTCGAGCTCACCAACTTGTAATGGAGGGATACAATTGGTGTCATG	989
PPP2CBY	....--............C...	978
PPP2CB	ATCGGAATGTGGTTACCATTTTCAGTGCACCCAATTACTGTTATCGTTGTGGGAACCAGG	1049
PPP2CBY	.................................-----------------------TTCC	1015
PPP2CB	CTGCTATCATGGAATTAGATGACACTTTAAAATATTCCTTCCTTCAGTTTGACCCAGCAC	1109
PPP2CBY	A...	1075

```
PPP2CB      CTCGTCGTGGAGAGCCTCATGTTACCCGGCGCACCCCAGACTACTTCCTGTAAATTTCTC   1169
PPP2CBY     .....G....................T.................................   1135

PPP2CB      CTGGGAAAAACTTGCCTTTGTATGTGGAAGTATACCTGGCTTTTTAAAA---TATATATA   1226
PPP2CBY     ...........A................................-...TATA........   1194

PPP2CB      TTTAAAAACAAAACAAAAAAAAGCAACAGTAATGTATGTGTTTCTGTAACAAATTGGATC   1286
PPP2CBY     ..............---...........................................   1251

PPP2CB      TGTCTTGGCATTAAACCATATCATGGACCAAAATGTGCCATACTAATGATGAGCATTTAG   1346
PPP2CBY     ....................................................A.......   1311

PPP2CB      CACAATTTGAGACTGAAATTTAGTACACTATGTTCTAGATCGGTCAGTCTTAACAGTTTG   1406
PPP2CBY     ........................................T...................   1371

PPP2CB      CCTGCTGTATTTGTAGTAACCATTTTCCTCTGGACTGTTCAAGCAAAAAAGGTAACTAAC   1466
PPP2CBY     .T......G..............................A..A.................   1431

PPP2CB      TCCTTCATCTTCTTTTGCACTTATTTGGAAATTTTAGTTATAGTGTTTAACTGGCATGGA   1526
PPP2CBY     ..........C.................................................   1491

PPP2CB      TTGATAGAGTTGGAGTTTTATTTTTAAGAAAAATTCACAAGCTAACTTCCATTTAATCCA   1586
PPP2CBY     ........C................................G..................   1551

PPP2CB      TTACCCTTTATTTTATTGAAATGTATAGTTAAATTAACTGAAGAAAAGATTCTTGGGAGT   1646
PPP2CBY     ....................G..................................A....   1611

PPP2CB      ATGTTGTCATAACATTTTAAAAGGTTTCCCTTCATTTAAACTAAATTACTGTTTTATGTT   1706
PPP2CBY     ............................................................   1671

PPP2CB      GATCTGCATATTTCTGTATATTTGTCATGACAGTGCTTGCATCCTATTTGGTGTACTGAG   1766
PPP2CBY     .............T...G.........---------..A.....................   1722

PPP2CB      CAAATAAACTTTCCATTTTAAACAAAA      1793
PPP2CBY     ............A..............      1749
```
