# Supplementary material for: Novel Y Chromosome Retrocopies in Canids Revealed through a Genome-Wide Association Study for Sex
Source: Genes (Basel). 2019 Apr 25;10(4):320. doi: 10.3390/genes10040320 (PMC6523286; doi:10.3390/genes10040320)
Supplement: Supplementary file 1 [file genes-10-00320-s001.zip › Supplementary figures/Fig S3.pdf]

Figure S3

```
Chr27   ACATCTTAAAGAGATTCAAGACCTGCAGAGTCGCCAGAAGCATGAAATTGAATCTTTGTA
ChrY    ---------...C......................G........................
ChrUn   ---------C..C...............................................

Chr27   TACCAAACTGGGAAAGGTTCCCCCTGCTGTCATTATCCCCCCAGCTGCCCCCCTTTCAGG
ChrY    ...............C...............................T..........A.
ChrUn   ............................................................

Chr27   GAGAAGAAGGCGACCCACTAAAAGCAAAGGCAGCAAGTCTAGTCGCAGCAGTTCCTTGGG
ChrY    ................C.....................................T.....
ChrUn   ..........T................................T................

Chr27   GAATAAAAGTCCCCAGCTTTCAGGTAACCTGTCTGGTCAGAGTGCTGCTTCAGTTTTGCA
ChrY    ............................................................
ChrUn   ............................T....................C.T........

Chr27   CGCTCAGCAGACCCTCCACCCTCCTGGCAACATCCCTGAGACTGGGCCAAATCAGCTGTT
ChrY    T............................................A..............
ChrUn   T.................................T.........................

Chr27   ACAGCCCCTTAAGCCATCTCCCTCCAGTGACAACCTCTACTCAGCCTTTACCAGTGATGG
ChrY    ...................A..............T..................AA.....
ChrUn   ..............T.............................................

Chr27   TGCCATTTCAGTCCCAAGCCTTTCTGCTCCAGGTCAAGGGACCAGCAGCACAAACACTGT
ChrY    ..................A...........T...................G.........
ChrUn   ............................................................

Chr27   CGGGGGAACAGTGAACAGCCAAGCTGCCCCAGCTCAGCCTCCTGCCATGACATCCAGCAG
ChrY    ....................................A.......................
ChrUn   .A........T.........................A.......................

Chr27   GAAGGGCACATTCACAGATGACCTACACAAGTTGGTAGACAATTGGGCCCGAGATGCCAT
ChrY    ............................................................
ChrUn   .................................A.....................C....

Chr27   GAATCTCTCAGGCAGAAGAGGAAGCAAAGGACACATGAATTATGAGGGCCCTGGAATGGC
ChrY    ................................................G...........
ChrUn   ............................................................

Chr27   AAGAAAGTTCTCCGCACCTGGTCAGCTGTGCATCTCCATGACCTCAAATCTGGGTGGTTC
ChrY    .............A....................C.T.......................
ChrUn   ............................................................

Chr27   TGCCCCCATCTCTGCAGCATCAGCTACCTCTCTAGGTCACTTCACCAAGTCTATGTGCCC
ChrY    ..........................................................T.
ChrUn   ...T................................................T......T

Chr27   CCCACAGCAGTACGGTTTTCCAGCTCCCCCCTTTGGCACTCAGTGGAGTGGGACGGGT-G
ChrY    ...........................A..........A.....A.........A...T.
ChrUn   ......................................................A...-.

Chr27   GCCCGGCACCACAGCCACTTGGCCAGTTCCAGCCTGTGGGAACTGCCTCCTTACAGAATT
ChrY    ...T...........T...................................C........
ChrUn   ............................................................
```

```
Chr27   TCAACATCAGCAATTTGCAGAAATCCATCAGCAACCCCCCAGGTTCCAACCTGCGGACCA
ChrY    ...................................G..........T..A..........
ChrUn   ......................................................A.....

Chr27   CTTAGACCTAGAGACTTTAACTGAGTAGATTTGGGGGCAGGAGATGGAATGCTGAGGGGT
ChrY    ............................................................
ChrUn   ............................G.......C.......................

Chr27   GGGTGGGAGGGGGGGGAATGGGAAAGTAGCCTATATACTAACTACTAGTGCTGCATTTAA
ChrY    ..............--............................................
ChrUn   ...............-...................................G........

Chr27   CTGGTTATTTCTTGCCAGAAAGGAATGTTTTTT
ChrY    T................................
ChrUn   T..........................A.....
```
