# Supplementary material for: Novel Y Chromosome Retrocopies in Canids Revealed through a Genome-Wide Association Study for Sex
Source: Genes (Basel). 2019 Apr 25;10(4):320. doi: 10.3390/genes10040320 (PMC6523286; doi:10.3390/genes10040320)
Supplement: Supplementary file 1 [file genes-10-00320-s001.zip › Supplementary figures/Table S1.pdf]

Table S1

Species	Common name	Sex	SRR#
Canis lupus familiaris	Dog	M	5190662
Canis lupus familiaris	Dog	M	5190661
Canis lupus familiaris	Dog	M	7956102
Canis lupus familiaris	Dog	F	5190660
Canis lupus familiaris	Dog	F	4011155
Canis latrans	Coyote	M	6324296
Canis latrans	Coyote	F	4029416
Lycaon pictus	African wild dog	M	2971425
Lycaon pictus	African wild dog	F	2971441
Vulpes vulpes	Red fox	M	5328110
Vulpes vulpes	Red fox	M	5328115
Vulpes vulpes	Red fox	F	5280489
Urocyon littoralis	Island fox	M	5198012
Urocyon littoralis	Island fox	F	5197998
Urocyon littoralis	Island fox	F	5198009
Urocyon littoralis	Island fox	F	5198001
